# Patch individual filter layers in CNNs to harness the spatial homogeneity of neuroimaging data

**DOI:** 10.1038/s41598-021-03785-9

**Published:** 2021-12-27

**Authors:** Fabian Eitel, Jan Philipp Albrecht, Martin Weygandt, Friedemann Paul, Kerstin Ritter

**Affiliations:** 1 Department of Psychiatry and Neurosciences | CCM, Berlin Center for Advanced Neuroimaging, Charité-Universitätsmedizin Berlin, Corporate Member of Freie Universität Berlin, Humboldt-Universität zu Berlin, and Berlin Institute of Health (BIH), 10117 Berlin, Germany; 2 Department of Neurology, NeuroCure Clinical Research Center, Experimental and Clinical Research Center, Max Delbrück Center for Molecular Medicine, Charité-Universitätsmedizin Berlin, Corporate Member of Freie Universität Berlin, Humboldt-Universität zu Berlin, and Berlin Institute of Health (BIH), 10117 Berlin, Germany; 3 Humboldt-Universität zu Berlin, 10117 Berlin, Germany; 4 Freie Universität Berlin, 14195 Berlin, Germany; 5 Bernstein Center for Computational Neuroscience, 10117 Berlin, Germany; 6 Einstein Center for Neurosciences Berlin, 10117 Berlin, Germany

**Keywords:** Computational neuroscience, Computer science

## Abstract

Convolutional neural networks (CNNs)—as a type of deep learning—have been specifically designed for highly heterogeneous data, such as natural images. Neuroimaging data, however, is comparably homogeneous due to (1) the uniform structure of the brain and (2) additional efforts to spatially normalize the data to a standard template using linear and non-linear transformations. To harness spatial homogeneity of neuroimaging data, we suggest here a new CNN architecture that combines the idea of hierarchical abstraction in CNNs with a prior on the spatial homogeneity of neuroimaging data. Whereas early layers are trained globally using standard convolutional layers, we introduce patch individual filters (PIF) for higher, more abstract layers. By learning filters in individual latent space patches without sharing weights, PIF layers can learn abstract features faster and specific to regions. We thoroughly evaluated PIF layers for three different tasks and data sets, namely sex classification on UK Biobank data, Alzheimer’s disease detection on ADNI data and multiple sclerosis detection on private hospital data, and compared it with two baseline models, a standard CNN and a patch-based CNN. We obtained two main results: First, CNNs using PIF layers converge consistently faster, measured in run time in seconds and number of iterations than both baseline models. Second, both the standard CNN and the PIF model outperformed the patch-based CNN in terms of balanced accuracy and receiver operating characteristic area under the curve (ROC AUC) with a maximal balanced accuracy (ROC AUC) of 94.21% (99.10%) for the sex classification task (PIF model), and 81.24% and 80.48% (88.89% and 87.35%) respectively for the Alzheimer’s disease and multiple sclerosis detection tasks (standard CNN model). In conclusion, we demonstrated that CNNs using PIF layers result in faster convergence while obtaining the same predictive performance as a standard CNN. To the best of our knowledge, this is the first study that introduces a prior in form of an inductive bias to harness spatial homogeneity of neuroimaging data.

## Introduction

In recent years, deep learning architectures relying on convolutional neural networks (CNNs) have advanced to a key technology for analyzing medical imaging data from various image sources including magnetic resonance imaging (MRI)^1–4^. In neuroimaging, state-of-the-art results have been achieved for diverse pixel-wise segmentation tasks (e.g., segmentation of white matter lesions, brain tumors or vessels)^5–8^ and image- or volume-wise classification of neurological or psychiatric diseases such as Alzheimer’s disease^9–11^, multiple sclerosis^12^ or schizophrenia^2^. The models used in most studies here are largely influenced by architectures which have been shown to be successful in computer vision tasks on natural images^1,4,13^.

**Figure 1:**
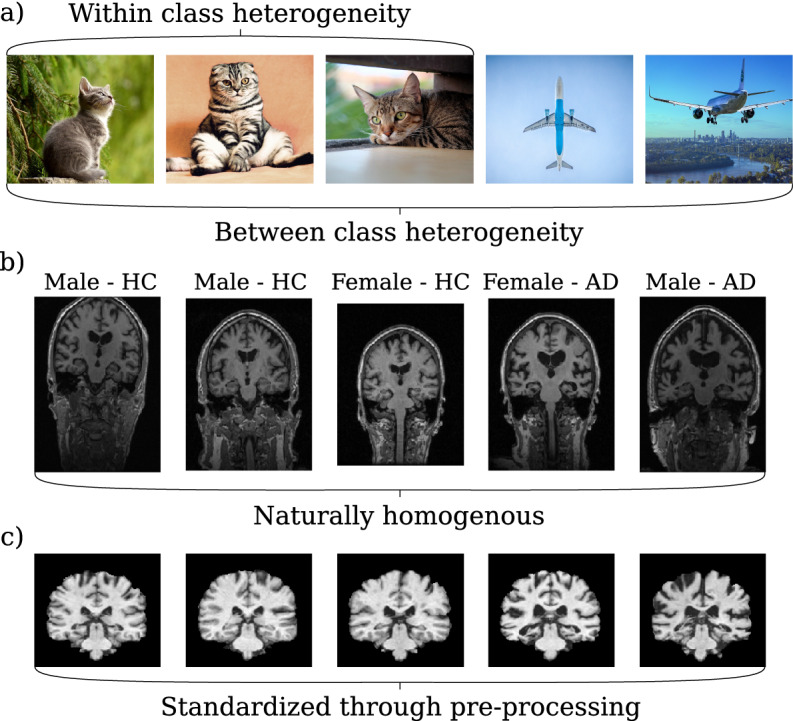
(**a**) Natural images are typically heterogeneous both within and between classes. (**b**) MR images of the human brain have homogeneous structures even among different sexes and between healthy subjects (HC) and diseased subjects (AD). (**c**) Through sophisticated pre-processing techniques, MR images are standardized to a common template reducing their variance further.

However, in contrast to natural images, neuroimaging data is much more homogeneous (see Fig. 1) and data sets are typically orders of magnitude smaller. The homogeneity of neuroimaging is due to (1) the inherent structure of the brain, which is mostly identical for individual subjects, i.e. each brain consists of the same parts (cerebellum, frontal lobe, occipital lobe, etc.), which are arranged in the same way (e.g., the occipital lobe is in the back); and (2) neuroimaging data is usually further homogenized by normalizing them to a shared template within the MNI space such as the ICBM 152 atlas^14–16^. For this, linear and/or non-linear transformations are used and different software packages are available (e.g., SPM^17^, FSL^18^ or ANTS^19^). Generally, this is done to ensure that a voxel at a certain location contains approximately the same brain region in every image and allows researchers to investigate a specific region (e.g., the hippocampus) across subjects. In particular, this is a major prerequisite for mass-univariate as well as multivariate pattern analysis, which have been extensively applied in the neuroimaging domain^20–23^. Acquiring large neuroimaging data sets has high requirements both financially and in terms of expertise (e.g. scanning protocol definition, safety measures, ethical guidelines), and the strict privacy regulations of medical data in many countries makes the publication of these data sets challenging. Therefore, many machine learning (ML) studies are carried out on rather small, local neuroimaging data sets, and results often do not generalize^24^.

In addition, training machine learning models on MRI data can be highly time-consuming. The 3-dimensional nature of MRI data and its high resolution leads to an extremely high feature count per sample. This causes model training for a single run to take up to several hours and on larger data sets up to several days. The training time is inflated again when performing cross-validation or repeated splitting of the data, as is typically recommended^25^. The large feature count can also require the data to be read from disk at training time if it exceeds the available memory, which further impacts training time. Furthermore, the longer a model trains, the more energy is being used, which increases its carbon footprint^26^. Hence, it has become a challenge to reduce the computation time of machine learning models and to improve their efficiency.

A common method to deal with small sample sizes and to reduce run time is to incorporate known information or assumptions about the data distribution into the learning model. Technically, this can be seen as introducing a prior. In neuroimaging-based disease classification and segmentation studies, priors have been incorporated into machine learning models by using extracted group-level features, topological structure in form of a probabilistic atlas, random elastic deformations, or other biophysical understandings^27–31^. However, the application of priors in CNN-based disease classification studies is not yet common, even though putting highly homogeneous data into standard CNN architectures is sub-optimal. This is because computer vision CNNs are optimized to deal with the high spatial variance of natural images (see Fig. 1a). By using weight-sharing, filters in both early and late layers are being optimized to capture signals regardless of their position. Were all images spatially standardized, i.e., objects were in the same position and had the same angle or viewpoint, it would suffice to search certain abstract objects, such as the ears of a cat, solely within a certain sub-space (i.e., a patch). Although it seems natural to exploit the spatial homogeneity of standardized MR images into a model prior, the technical integration of priors into CNNs is difficult and, to the best of our knowledge, has not been done yet.

**Figure 2:**
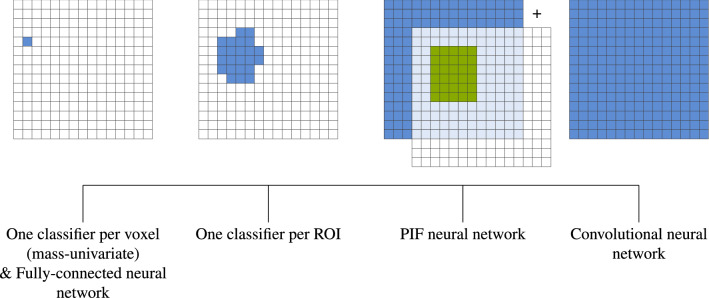
Comparison of the number of voxels each feature/kernel uses per model. The grid shows the entire input and in blue/green how much of the input is used in the respective models. Mass-univariate studies use a single voxel per classifier, fully-connected neural networks also use a single weight per voxel albeit combining them after. ROI-based models typically train a single classifier based on an entire ROI or extract a single feature from an ROI. Patch individual filter (PIF) neural networks use both the entire input for lower level features and patches for higher level (latent) features (shown in green). CNN filters use the entire input of each layer throughout the entire network (under some conditions regarding stride and dilation).

Another method, aimed at improving learning in small sample size regimes and reducing its training time, is to reduce the number of features through selection^32^. A model parameter, such as a neural network filter, that is being trained on the entire input will be subject to a greater superimposition of different distributions (from signal and noise) than it would on a smaller selection of those features, i.e. a sub-space. If we can assume that most selected sub-spaces contain sufficient discriminatory information, then the disentanglement task on each sub-space becomes easier. Therefore, training model parameters on a sub-space of the input should require fewer training samples and iterations for convergence. In Fig. 2, we plot typical neuroimaging analysis methods which use non-data-driven feature selection on the spectrum of how many input features each filter or classifier uses. The extreme case of reducing the input would be to fit a model for each voxel individually. This is the case in mass-univariate studies and entails the multiple comparison problem^33–35^. A fully-connected neural network is similar in that the weights are learned based on a single input feature and neighboring information is lost. An intermediate solution would be to train models on regions-of-interest (ROIs) or, more generally, image patches^36,37^.

**Figure 3:**
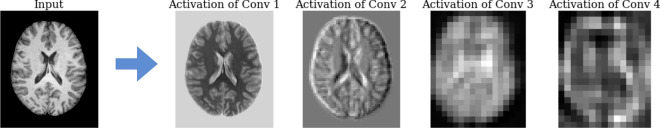
Latent activation maps of convolutional layers preserve the spatial representation of the input.

In this study, we combine a spatial homogeneity prior with feature selection by introducing a new CNN architecture relying on patch individual filter (PIF) layers. In contrast to standard convolutional layers, PIF layers do not perform weight sharing across the entire input but learn individual filters for each location in the data. Since we assume that individual filters are especially relevant for more abstract features, we only exchanged later layers with PIF layers. For early layers, we used standard convolutional layers to learn globally relevant low-level features such as edges and blobs. Therefore, PIF layers invoke feature selection in the latent space instead of the input space. In Fig. 3, we show that a CNN retains the broader spatial specificity of the input space along the convolutional layers, therefore splitting in the latent space is analogous to splitting in the input space.

We evaluated the PIF-architecture with respect to a baseline vanilla CNN-architecture, and a patch-based CNN architecture for three exemplary tasks within the neuroimaging domain; namely sex classification based on the UK Biobank imaging data^38^, Alzheimer’s disease (AD) detection based on the Alzheimer’s Disease Neuroimaging Initiative^39^ (ADNI) database and multiple sclerosis (MS) detection based on private data from Charité-Universitätsmedizin Berlin. We hypothesized that PIF layers should increase predictive performance in smaller data sets and reduce training time for all kinds of data sets. In all cases, the PIF architecture resulted in a faster convergence, measured by the time until early stopping occurred, while obtaining areas under the receiver operating characteristic curves (AUC ROC) that are statistically not separable from the simple baselines.

## Related work

PIF layers can be understood as a generalization of local convolutions where the sum of patch size and padding size equals the kernel size (e.g., implemented in Lasagne https://lasagne.readthedocs.io/en/latest/modules/layers/local.html and Keras https://keras.io/layers/local/). In patch-based training^5,40,41^, multiple patches are sampled from the data set and fed into the same classifier regardless of the position of each patch. Since the filters of the classifier are applied on all patches, the weights are shared between patches. Conversely, within PIF layers, weights are only shared within a spatially restricted patch. PIF layers are similar to the splitting of layers in Split-CNN^42^. Split-CNN splits both the input and feature maps into patches to reduce memory usage of the network. In contrast to PIF layers, Split-CNN can split using stochastic patch locations but tends to degrade predictive performance in most cases. PIF layers are furthermore different from PatchGANs^43,44^ which use Markovian patches as input for a discriminator network to focus penalization on high-frequency structures. Another approach introduced in Kamyar et al.^45^ uses a greedy two-stage training strategy: first, a patch-wise model is trained, second, the input image is split into 12 patches and latent features of the first model are extracted, and lastly, those feature maps are concatenated to train a final classification network. Since the extracted feature maps are concatenated in order to create a spatially smaller 3D input for the classification network, weights are in turn again shared between the feature map patches. No other method uses the abstraction of neural networks to spatially restrict the weight sharing in later layers only.

## Methods

### Priors in machine learning

Priors are assumptions about the world which shape how a model learns. In a way, priors are similar to data but are used differently: “[...] any additional information beyond the immediate data $D$ of the current problem is by definition ‘prior information’ ”^46^. In Bayesian learning, the Bayes Theorem

$$\begin{aligned} P(H|D) = \frac{P(D|H)}{P(D)}P(H) \end{aligned}$$

determines the probability of an event using a prior distribution $P(H)$ together with the likelihood $P(D|H)$ divided by the marginal distribution of the data $P(D)$. In neuroimaging, an example is determining the probability that a person has Alzheimer’s disease given their MR image (posterior probability $P(H|D)$). We know a priori the incidence rate of the disease $H$ in our society or data set ($P(H)$) and estimate the likelihood that someone shows signs of AD such as atrophy $D$ in their MRI ($P(D|H)$), as well as the probability of having atrophy regardless of their diagnosis ($P(D)$). In deep learning, priors or inductive biases can be specific choices in the network design that are based on assumptions about the task. Common examples include the use of convolutions for enabling equivariance over space or recurrence for equivariance over time^47^. Here, the data is being used to train a model, while the prior is being used to define *how* to learn from the data. If the prior makes correct assumptions about the world, it might compensate for too little data and facilitate faster training.

### Description of PIF layers

For the analysis of spatially homogeneous and normalized MRI data, we introduce in this section a new CNN architecture relying on PIF layers. Although we perform all experiments in 3D, we describe and visualize here the methods for simplicity in 2D. PIF layers consist of 3 stages: (i) split, (ii) process and (iii) reassemble. Each output feature map of the previous layer is first split (i) into patches of size $(s_{x} \times s_{y})$. Next, the patches $p_{ij}$ centered at row $i$ and column $j$ of all feature maps are processed (ii) with a series of local convolutions of kernel size $(k_{x} \times k_{y})$.

**Figure 4:**
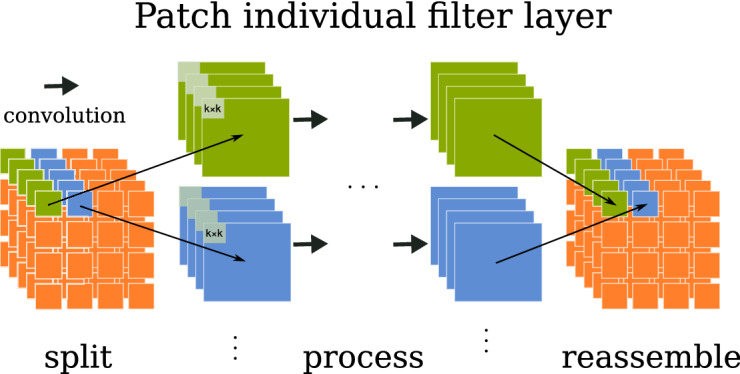
Depiction of a patch individual filter (PIF) layer in 2D. In this setting, inputs are 5 feature maps from a previous layer. Each feature map is being split into 16 patches and convolutions are applied patch-wise. Finally, the feature maps are reassembled in the same order.

In comparison to the convolution operation

1 $$\begin{aligned} z = \sum _{m} \sum _{n} I(m,n)K(i-m,j-n), \end{aligned}$$

in which a kernel $K$ is convolved with an input $I$, the PIF operation

2 $$\begin{aligned} z_p = \sum _{{\hat{m}}} \sum _{{\hat{n}}} I({\hat{m}},{\hat{n}})K_p(i-{\hat{m}},j-{\hat{n}})\, \forall p \in P \end{aligned}$$

applies a patch specific kernel $K_p$ to the current patch $p$, where ${\hat{m}}, {\hat{n}} \in p$ and $P$ is the set of all patches $p$. When $s > k$, weights are shared within each patch $p_{ij}$ but not across patches. Lastly, all patches are reassembled (iii) in the same order as they were split. Figure 4 shows an overview of the layer design. PIF layers can be easily integrated into many CNN architectures and can be modified to contain other layer types besides convolutions. An implementation using PyTorch can be found here: https://github.com/derEitel/patch_individual_filter_layer.

When splitting a feature map into patches, one creates artificial borders which could reduce training performance. Each patch has several new and unnatural borders. These borders potentially cut through objects that the network might learn as a whole. For example, the splitting could cause a feature map region representing the hippocampus to be split into two patches. The first downside is that this leads to potential border effects in areas that would normally not be affected. Second, a symptom such as hippocampal atrophy might only be visible in one of the patches, causing the two patches to disagree. Simply speaking, one patch might forward activations that support the disease class, while the other patch might inhibit activation, supporting the control class. To mitigate these issues we perform a parallel strain of network in which the patches are split with an overlap to the original split. Each original patch location $(x_o, y_o)$ is shifted by half its patch size to the overlapping location $(x_{ov}, y_{ov})$:

3 $$\begin{aligned} (x_{ov}, y_{ov}) = (x_{o} + \frac{s_{x}}{2}, y_{o} + \frac{s_{y}}{2}). \end{aligned}$$

This way, patches are added in a minimalistic fashion, centralizing the overlap between existing patches while neglecting additional patches at the image borders that would require padding and are likely less informative. We train all patches by averaging the gradients between the overlapping and non-overlapping patches during the backward pass.

### LRP visualization

Layer-wise relevance propagation (LRP) is a method to analyze the behavior of deep neural networks and other machine learning methods^48^. It has been used in several studies in MR imaging^49–51^ and it was shown that identified relevant regions can overlap with clinically established relevant brain regions such as the medial temporal lobe in Alzheimer’s disease^11,52^ and the corpus callosum in multiple sclerosis^12^. LRP uses backpropagation to transfer the output score of the network into the input space and therefore creates heatmaps that show the relevance of each pixel. We propagate the relevance following the $\alpha$/$\beta$-rule:

4 $$\begin{aligned} R_{i}^{l} = \sum _j \left( \alpha \frac{z_{ij}^{+}}{\sum _k z_{kj}^{+}} - \beta \frac{z_{ij}^{-}}{\sum _k z_{kj}^{-}} \right) R_{j}^{l+1}. \end{aligned}$$

here, the relevance from layer $$R^{l+1}$$ is backpropagated to its preceding layer $$R^{l}$$. Activations are divided into positive and negative contributions ($$z^{+/-}_{ij})$$ between nodes $$i$$ and $$j$$. Additionally, activations are normalized with the sum of the positive/negative activations from that layer. The hyperparameters $$\alpha$$ and $$\beta$$ need to be tuned and are confined to $$\alpha = 1 + \beta$$. In this study, we set $$\beta = 4$$ which we found to work well in practice. Typically, one invokes the LRP backpropagation with the activation of the final layer, here we furthermore start with the activation of hidden neurons to obtain the relevance of a specific filter within the network. The adaptation of the LRP algorithm to the PIF layers requires splitting of the relevance in the same manner as we split the gradients during training. The relevance from the overlapping patches is simply averaged with the relevance from the non-overlapping patches.

## Experiments

### Model architecture

**Figure 5:**
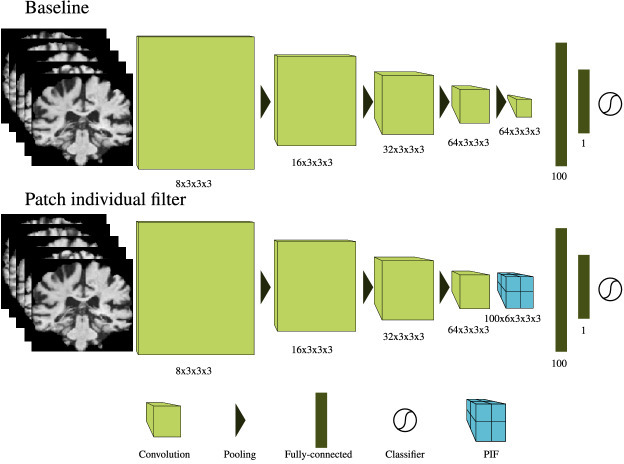
Overview of the CNN architecture, the top row shows the baseline which has been optimized for the respective task. The bottom row shows the same architecture with the last convolutional and pooling layer replaced by a PIF layer. Written below each convolutional layer are the number of filters and their size and below each fully-connected layer is the number of output neurons. Below the PIF layer the number of patches, number of convolutions per patch, and size of convolution kernels are displayed. Shown are model A and model A-PIF as used on the UK Biobank.

Based on the theoretical motivation, we compare here a baseline CNN model, whose architecture was optimized for the given task, to the same CNN architecture in which the final convolutional layer (before fully-connected layers) was replaced with a PIF layer (see Fig. 5). The model architecture is a shallow VGG-inspired^53^ CNN which contains only convolutional, max pooling, and fully-connected layers. Two variants, model A and model B, with 4 or 5 convolutional layers, as well as differences in their number of filters were used to test the effect of different model capacities. By using two architecture settings we avoid mistaking the effects of feature count for the effect of the PIF layer itself. In our experiments, on each data split, both the baseline and the PIF model were given a higher, a lower as well as an approximately even feature count. While the general architecture is the same for all data sets, slight modifications were necessary to match the dimensionality of the data. The deviations from Fig. 5 for each data set are depicted in the Supplementary Appendix. Additionally, we have tested a patch-based architecture which we adapted from Liu et al.^54^. The patch-based architecture in Liu et al.^54^ contains 4 convolutional layers and 2 fully-connected layers. Here, we apply it on patches obtained by splitting the input image into 2 $\times$ 3 $\times$ 2 patches across the x, y, and z dimensions. The exact implementation of all experiments can be found here: https://github.com/derEitel/patch_individual_filter_layer.

### Data augmentation and transformation

For data augmentation, we used translation and flipping along the sagittal axis, which are typical augmentations in neuroimaging^55–57^. We applied data augmentation only to the UK Biobank and the ADNI data, but not to the MS data set, since here the validation performance was reduced when using data augmentation. For the PIF architecture, we performed only translation and not flipping, since the PIF layer requires patches to have the same content during each training iteration. All images were intensity normalized by dividing each by its maximum value before training.

### Validation

The models were compared using both their predictive performance and their convergence speed. The predictive performance was measured using the area under the receiver operating characteristic curve (AUC ROC), balanced accuracy, sensitivity, and specificity. To evaluate the hypothesis that PIF architectures require fewer training iterations, we measured the number of iterations until early stopping occurs. Early stopping is a kind of regularization, which forces the model to end training after the performance has not improved for a fixed amount of iterations. Early stopping can also be caused by a model not being able to leave the initial optimization basin or being stuck in a poor local minimum, hence using the number of early stopping iterations as a measure of convergence is only feasible when the model achieves a good predictive performance. Furthermore, we investigated the training time of each experiment in seconds to measure its efficiency. All models have been run on the same computing system to avoid hardware-based effects on the training time.

As the results of CNNs can considerably vary between single training runs on neuroimaging data^56^, we run each experiment several times. Specifically, we follow the recommendations in Varoquaux et al.^25^ and repeat our experiments in 10 outer folds, each time randomly sampling the data into training, validation, and test sets. For each of the 10 data splits, we run hyperparameter optimization on the validation set by performing 5 experiments with randomly sampled hyperparameters from Table 1. We defined that both architecture variants needed to be sampled at least two times within five repetitions, whereas all other parameters had no sampling constraints. The best hyperparameter settings per validation set are then used on the independent test set, leading to 10 test scores which were averaged to present the final model performance. In total, this leads to training each model 50 times. As the run time on the full UK Biobank data set is prohibitively long, two instead of 10 random data splits were used on that data set.

**Table 1:** List of hyperparameters that were randomly sampled from. See Supplementary Appendix A for the model architectures.

Hyperparameter	Values
Architecture	[Model A, Model B]
Learning rate	[$1 \times 10^{-4}$, $5 \times 10^{-5}$]
Mini-batch size	[4, 6, 12]
Patience	[12, 16]

### Data sets

To study the effect of PIF layers we have compared the performance on three different structural MRI data sets. As we hypothesize that PIF layers should need fewer training examples to learn relevant features, we have run an additional comparison on a randomly sampled 20% subset of the selected UK Biobank and ADNI data sets. As the number of samples for the MS data set is already small, we did not use a subset here. All methods were carried out in accordance with relevant guidelines and regulations.

#### UK Biobank

For this study, 1854 T1-weighted images (MPRAGE, 3 Tesla) from the UK Biobank^38^ (www.ukbiobank.ac.uk) were randomly chosen to train a sex detection classifier. The MR images were obtained pre-processed from the UK Biobank repository combining data from several sites and scanners. The pre-processing pipeline included defacing, reduction of the field of view to remove empty space around the brain, and gradient distortion correction. Furthermore, images were non-linearly transformed to MNI152 space using the FMRIB Software Library^18^ (FSL). The final image size is 182 $\times$ 218 $\times$ 182 voxels. The target of the trained classifiers is to distinguish between female (n = 1005) and male (n = 849) brains. Data was split for each of the 10 repetitions into separate test (20%), validation (16%), and training (64%) sets.

#### ADNI

969 T1-weighted images (MPRAGE, 1.5 Tesla) from the Alzheimer’s Disease Neuroimaging Initiative^39^ (ADNI; www.adni.loni.usc.edu) database were used to discriminate subjects with Alzheimer’s disease (AD) from healthy controls (HCs). The ADNI was launched in 2003 as a public-private partnership, led by Principal Investigator Michael W. Weiner, MD. The primary goal of ADNI has been to test whether serial magnetic resonance imaging (MRI), positron emission tomography (PET), other biological markers, and clinical and neuropsychological assessment can be combined to measure the progression of mild cognitive impairment (MCI) and early Alzheimer’s disease (AD). The MR images come from different sites and scanners and were downloaded partially pre-processed. Already applied pre-processing steps included corrections for gradient non-linearity, intensity inhomogeneity, and phantom-based distortion. We furthermore registered all images to the ICBM152 standard template (asymmetric version 2009c at 1 mm) using non-linear registration from the Advanced Normalization Tools^19^ (ANTs). The final image size has been reduced to 96 $\times$ 114 $\times$ 96. The images stem from 193 AD patients and 151 HCs with up to three time points. To avoid data leakage, splitting of the data set for each repetition was done on the patient level and not on the image level leading to disjoint test (18%), validation (10%), and training (72%) sets.

#### VIMS

147 fluid-attenuated inversion recovery (FLAIR, 3 Tesla) images from the VIMS study (https://neurocure.de/en/clinical-center/clinical-studies/current-studies.html) of the NeuroCure center at Charité-Universitätsmedizin Berlin were used to separate patients with relapse-remitting multiple sclerosis (MS) and healthy controls (please see also Eitel et al.^12^). All images were acquired from the same 3T scanner (Tim Trio Siemens, Erlangen, Germany). After bias-field correction and robust field of view selection, the corresponding MPRAGE sequences were linearly registered to MNI space using FSL^18^. FLAIR images were then co-registered to the MPRAGE images using spline interpolation. The final image size has been reduced to 96 $\times$ 114 $\times$ 96. 76 images stem from patients with relapsing-remitting multiple sclerosis (MS) according to the 2010 McDonald criteria^58^, and the remaining 71 images stem from healthy controls. As the data set is small we dedicated a larger portion to the training set leading to splits of 15% for testing, 8.5% validation, and 76.5% training in each repetition.

## Results and discussion

Table 2 shows the predictive performance results from all models averaged over 10 randomly selected test sets (2 in case of the large UK Biobank data set). The predictive performance between baseline and PIF model are almost identical for most metrics. For example, the AUC ROC on the full UK Biobank data (baseline 98.81%; PIF 99.10%) and on the full ADNI data set (baseline 88.89%; PIF 86.88%) differ only slightly, with the leading algorithm switching from experiment to experiment. This shows that the introduction of the PIF layer does not alter the predictive performance of the CNN. Otherwise, both baseline and PIF architecture strongly outperformed the patch-based approach in almost all metrics. The ROC curves are shown in Fig. 7 and Supplementary Fig. 2 in the Appendix. All results of the baseline and PIF model are comparable to those obtained in the current literature (sex classification^59–61^; AD detection^62^; MS detection^12^), however due to the large impact of different data sets, data splits, weight initialization and other factors of variance^63^ a direct comparison of accuracies between studies has little meaning. As noted in Wen et al.^62^ shallow models consisting of only convolutional and fully-connected layers are usually sufficient for classification on MR images and the exact number of layers has less influence than other factors of variation. We similarly found that neither model configuration A nor B was chosen more often than the other across all experiments.

Based on our results, we reject the hypothesis that PIF layers reduce the number of samples required to train a model. While we were expecting similar predictive performances between baseline and PIF model on the large data sets, we were expecting better predictive performances on the small data sets. However, in contrast to other work utilizing the splitting of feature maps^42^, we did not find a degradation of predictive performance either.

**Figure 6:**
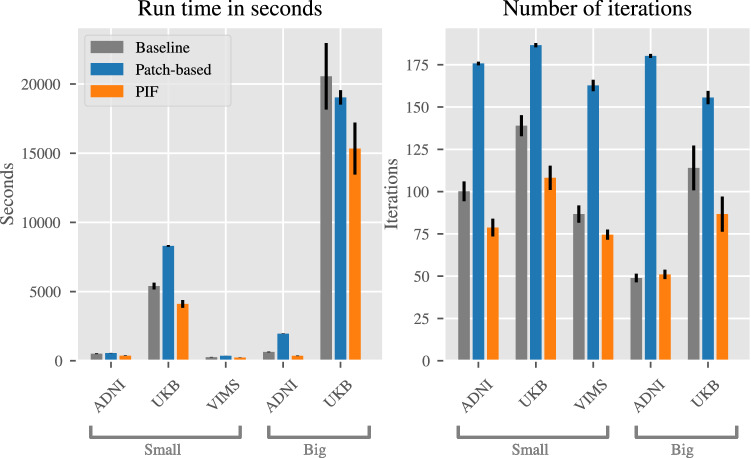
Training time for all runs in seconds and number of iterations. Error bars depict standard error.

Figure 6 shows the training time of all experiments both in seconds and the number of iterations. Here, we compare all 50 runs (10 for the large UK Biobank data set) in terms of their training time since we are interested in reducing the overall time to train a model. In all experiments we can see that the PIF model strongly outperforms both patch-based and baseline models. Notably, on the ADNI small data set the PIF layer reduces the training time by almost 28% from 511.42 to 369.16 s and on the large UK Biobank data set by 25% from 20577.2 to 15331.8 s in comparison to the baseline.

For the number of iterations, the difference between baseline and patch-based approach was also highly apparent. On all but the ADNI big data set the average number of iterations until convergence has dropped largely by up to 24%. The number of iterations required for the patch-based model to converge is much larger than both baseline and PIF model on all data sets.

**Table 2:** Results of the binary classification tasks. Area under the receiver-operating curve, sensitivity, specificity, balanced accuracy are reported as averages over 10 repetitions. Standard deviation is reported in parentheses.

Data	Model	AUC ROC	Sens.	Spec.	Bal. acc.
**Large data sets**					
UK Biobank	Baseline	98.81% (0.21)	96.62% (0.16)	90.66% (0.42)	93.64% (0.13)
UK Biobank	Patch-based	79.41% (2.41)	66.11% (1.06)	77.40% (9.26)	71.75% (4.10)
UK Biobank	PIF	99.10% (0.27)	97.78% (1.49)	90.64% (0.26)	94.21% (0.61)
ADNI	Baseline	88.89% (6.51)	84.09% (7.34)	78.75% (11.43)	81.42% (7.48)
ADNI	Patch-based	78.23% (6.33)	67.23% (12.02)	71.23% (13.89)	69.23% (6.70)
ADNI	PIF	86.88% (5.92)	79.19% (8.45)	77.49% (9.68)	78.34% (6.98)
**Small data sets**					
UK Biobank	Baseline	94.30% (3.31)	84.93% (10.90)	86.13% (5.98)	85.53% (4.69)
UK Biobank	Patch-based	65.31% (5.00)	44.16% (33.93)	66.32% (29.83)	55.24 % (4.78)
UK Biobank	PIF	93.99% (2.92)	91.93% (5.48)	81.33% (6.26)	86.63% (4.68)
ADNI	Baseline	85.78% (7.42)	77.22% (7.92)	77.80% (10.88)	77.51% (6.88)
ADNI	Patch-based	49.77% (15.33)	60.26% (51.31)	40.21% (51.46)	50.23% (0.50)
ADNI	PIF	86.74% (7.91)	75.31% (10.81)	79.93% (10.78)	77.62% (7.29)
MS	Baseline	87.35% (8.76)	79.73% (17.24)	81.23% (15.07)	80.48% (10.24)
MS	Patch-based	81.00% (8.30)	100.00% (0.)	19.96% (13.32)	59.98% (6.66)
MS	PIF	86.22% (8.10)	74.38% (10.61)	81.27% (17.61)	77.83% (9.67)

Increasing the resource efficiency of neural networks is an important challenge in order to reduce their $CO_2$ footprint and enable a wider spread of research into departments with tight limitations on expensive computing resources such as GPUs^26^. Similarly, Sze et al.^64^ stated that “techniques that enable efficient processing of DNNs [deep neural networks] to improve energy efficiency and throughput without sacrificing application accuracy or increasing hardware costs are critical to the wide deployment of DNNs in AI systems”. By using PIF layers researchers applying deep learning in neuroscience can reduce their model training time by up to 28%.

### Heatmap analysis

We created several LRP heatmaps to support the motivation for PIF layers and determine differences caused by the addition of a PIF layer. Since the UK Biobank data set has the largest sample size and both baseline and the proposed architecture achieved a high, very similar performance, we only show visualizations for the UK Biobank data. For evaluation, a high predictive performance is important, while differences between the models’ performances should be small. Otherwise, it remains unclear whether differences come from a gap in performance or the architecture itself. The figures shown are based on the selected models after hyperparameter search from the first outer fold, which was run on the test set.

**Figure 7:**
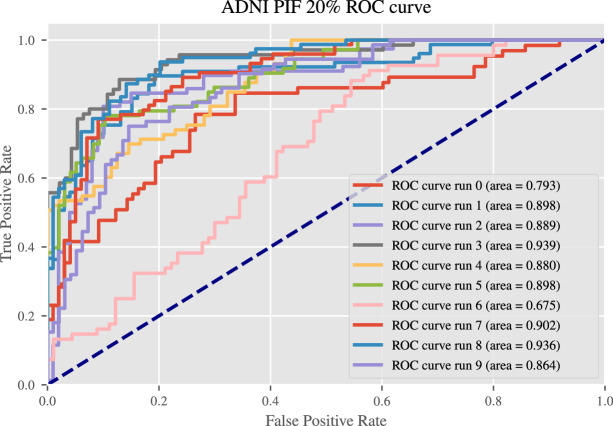
Receiver operating characteristic (ROC) curves for all 10 runs of the PIF model trained on the 20% ADNI data set.

**Figure 8:**
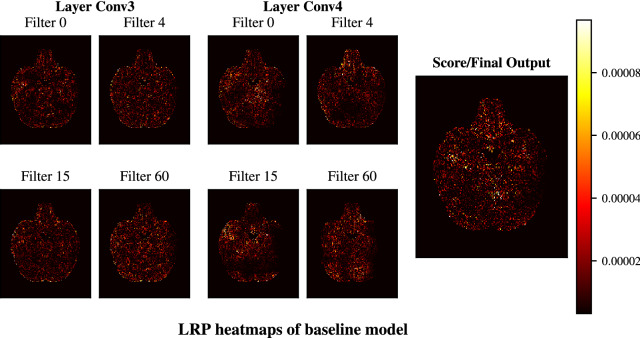
Heatmaps of the baseline trained on the big UK Biobank data set generated from the last two convolutional layers and the final output. Four filters from the convolutional layers were randomly selected. Note that there is no special relationship between the filters at the same location (i.e. filter 0 at conv 3 and conv 4) as each filter is applied to all previous feature maps.

First, we used the baseline model to investigate whether higher layer features in a CNN trained on MRI data will have a more localized focus than features from lower layers do. We generated LRP heatmaps using the outputs of both the intermediary and the final output layer (Fig. 7). Figure 8 shows the heatmaps obtained by backpropagating the activations of 4 randomly selected filters in convolutional layers 3 and 4 as well as the heatmap of the model output. The comparison between the heatmaps of convolutional layers 3 and 4 shows that the lower layer has more connected and dense heatmaps across all shown filters whereas the higher layers have more sparse heatmaps with several regions showing no activity, such as the right inferior and medial temporal lobes in filters 60 and 15 and parts of the cerebellum in filters 4 and 15. The LRP heatmap of the model score on the other hand does not portray these empty regions. As the final output is a combination of all convolutional filters the heatmap becomes more holistic and dense again. Nevertheless, trends emerge in many layer 5 filters which are reflected in the final output, such as a lower focus on the edge of the right inferior temporal lobe. This shows that the filters of the last convolutional layer tend to be more locally specific in the baseline architecture. As the PIF architecture enforces this higher layer locality through its feature map patches, it could be one of the causes of its faster convergence over the baseline.

**Figure 9:**
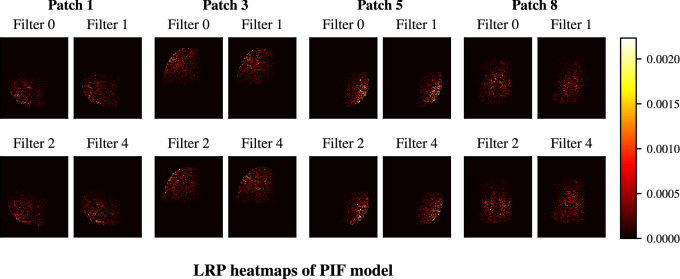
LRP heatmaps of the PIF model trained on the big UK Biobank data set using the PIF layer output to generate patch and filter specific heatmaps. Each patch learns individual filters and therefore patches at the same location (i.e. filter 0 across patches) do not share a specific relation.

**Figure 10:**
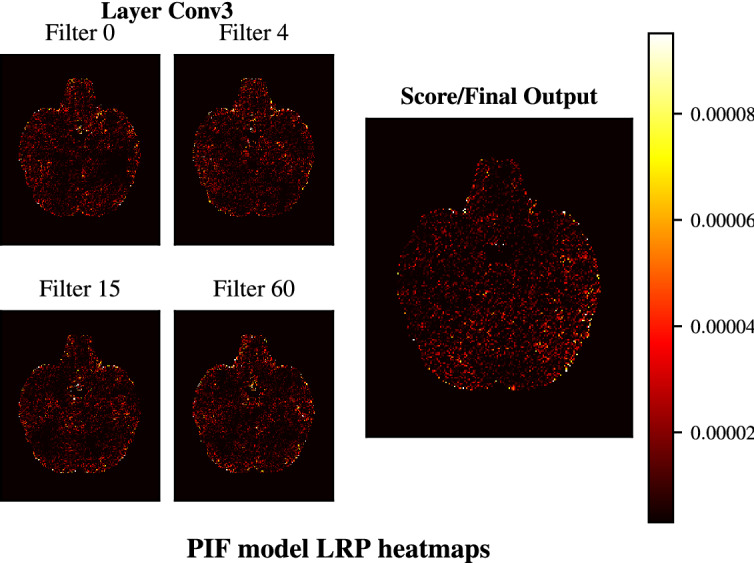
LRP heatmaps of the PIF model trained on the big UK Biobank data set based on layer 3 feature maps (the final layer before the PIF layer) and the model output (score).

Next, we compared heatmaps from the baseline model to the proposed PIF architecture. Figure 9 shows the heatmaps of the UK Biobank PIF architecture, obtained from the PIF layer. Here, the locality due to the patches is highly apparent. Patches 1, 3, and 5 represent different quadrants, whereas patch 8 is an overlapping patch that is in the center of the image. Beyond that, the layer 4 heatmaps of the PIF model, as depicted in Fig. 10, show that the sparseness occurring in baseline layer 4 does not simply move down to layer 3 in the PIF model. If this had been the case, it could have been an indicator that the PIF layer does not add any disentanglement value and that the remaining model is able to learn similar features with a smaller capacity. Here, we can rather see that the layer 3 heatmaps between baseline and PIF model seem to be very similar in terms of general structure, indicating that the PIF layer does not obstruct the general learning performance of the CNN. Finally, the LRP heatmap of the final output in Fig. 10 shows a strong resemblance to the baseline heatmap in Fig. 8, although it is slightly less noisy.

### Limitations

The major limitation of PIF layers is that it requires all examples to be spatially standardized. By design PIF layers require spatial homogeneity and ideally registration, while all inputs need to have the same number of features per dimension (e.g. same number of voxels). As the pre-processing was optimized for each data set individually, this refrained us from training a single classifier on all presented tasks. Nevertheless, when MRI data, as well as pre-processing pipelines, become more and more standardized, a holistic architecture might become conceivable. Furthermore, we have recently shown that linear and non-linear pre-processing of structural MRI increases the balanced accuracy on AD detection by 6–7% using a vanilla CNN^65^. This shows that the lengthy pre-processing process, which we assume a requirement for PIF layers, is also vital for achieving competitive results with other CNN architectures and henceforth, we have not considered the pre-processing times in this study. Potential border effects pose another limitation. While we reduced the risk of splitting important objects by using overlapping patches, we thereby introduced additional artificial borders which might be unfavorable and their effect should be addressed in future studies. Next, due to many ways to pre-process and split the data as well as large hyperparameter spaces, it is generally difficult to compare different deep learning algorithms, and thus we can not rule out that better configurations for each of the tasks exist. To keep the comparison as simple as possible, we only used randomly initialized CNN baselines. Lastly, backpropagation-based attribution methods such as LRP, which we have used to produce heatmaps in Figs. 8, 9 and 10, have lately been criticized in their ability to show relevant features about the given task^66,67^. However, in the setting of this study, the heatmaps were not used to determine causal relationships in the input–output mapping but solely to investigate the locality of different layers. Future studies might investigate the effect of transfer learning, combining several modalities and other validation strategies.

## Conclusion

In this study, we have introduced PIF layers for CNNs. Based on the understanding that higher level layers learn more abstract and localized features, we have reinforced that learning direction by splitting higher level feature maps into patches and learning CNN features without weight-sharing between those patches. In scenarios where data is naturally homogeneous or spatially normalized, PIF layers can be introduced in order to reduce training time and the number of iterations until model convergence. PIF layers can be used in the same way as convolutional layers and do not degrade model performance, unlike other splitting methods^42^. They can be seen as introducing a spatial prior into the neural network model. Based on further knowledge about the data, one could tune this prior by adjusting the patch size to the size of a certain biomarker or relevant sub-regions in an image or could weigh patches based on a pre-defined hypothesis. Potential future applications are other standardized medical sets, e.g., coming from other modalities (PET, CT, other MRI sequences, etc.) or other parts of the body also requiring normalization. Tasks that require even more regional specificity, such as segmentation, might profit from the application of PIF layers as well.

## Supplementary Information

Supplementary Information.
