# Supplementary material for: Patch individual filter layers in CNNs to harness the spatial homogeneity of neuroimaging data
Source: Sci Rep. 2021 Dec 27;11:24447. doi: 10.1038/s41598-021-03785-9 (PMC8712523; doi:10.1038/s41598-021-03785-9)
Supplement: Supplementary file 1 — Supplementary Information. [file 41598_2021_3785_MOESM1_ESM.pdf]

Appendix

VIMS

Model A	#Filters	Size	Stride	Input Size	Model A PIF	#Filters	Size	Stride	Input Size	Model B	#Filters	Size	Stride	Input Size	Model B PIF	#Filters	Size	Stride	Input Size
Conv	16	3	1	96, 114, 96	Conv	16	3	1	96, 114, 96	Conv	64	3	1	96, 114, 96	Conv	64	3	1	96, 114, 96
ELU					ELU					ELU					ELU				
Max Pool		3	3	94, 112, 94	Max Pool		3	3	94, 112, 94	Max Pool		3	3	94, 112, 94	Max Pool		3	3	94, 112, 94
Conv	32	3	1	31, 37, 31	Conv	32	3	1	31, 37, 31	Conv	64	3	1	31, 37, 31	Conv	64	3	1	31, 37, 31
ELU					ELU					ELU					ELU				
Max Pool		3	3	29, 35, 29	Max Pool		3	2	29, 35, 29	Max Pool		3	3	29, 35, 29	Max Pool		3	3	29, 35, 29
Conv	64	3	1	9, 11, 9	Conv	64	3	1	14, 17, 14	Conv	64	3	1	9, 11, 9	Conv	64	3	1	10, 12, 10
ELU					ELU					ELU					ELU				
Conv	64	3	1	7, 9, 7	Conv	64	3	1	12, 15, 12	Conv	64	3	1	7, 9, 7	PIF 5x5x5	4	3	1	10, 12, 10
ELU					ELU					ELU					ELU				
Max Pool		3	1	5, 7, 5	PIF 5x5x5	4	3	1	10, 13, 10	Max Pool		3	1	5, 7, 5	Linear		100		1512
Linear		100		2880	ELU					Linear		100		2880	ELU				
ELU					Linear		100		1512	ELU					Linear		1		100
Linear		1		100	ELU					Linear		1		100					
					Linear		1		100										

UKBiobank

Model A	#Filters	Size	Stride	Input Size	Model A PIF	#Filters	Size	Stride	Input Size	Model B	#Filters	Size	Stride	Input Size	Model B PIF	#Filters	Size	Stride	Input Size
Conv	8	3	1	182, 218, 182	Conv	8	3	1	182, 218, 182	Conv	8	3	1	182, 218, 182	Conv	8	3	1	182, 218, 182
ELU					ELU					ELU					ELU				
Max Pool		3	3	180, 216, 180	Max Pool		3	3	180, 216, 180	Max Pool		3	3	180, 216, 180	Max Pool		3	3	180, 216, 180
Conv	16	3	1	60, 72, 60	Conv	16	3	1	60, 72, 60	Conv	32	3	1	60, 72, 60	Conv	32	3	1	60, 72, 60
ELU					ELU					ELU					ELU				
Max Pool		3	3	58, 70, 58	Max Pool		3	3	58, 70, 58	Max Pool		3	3	58, 70, 58	Max Pool		3	3	58, 70, 58
Conv	32	3	1	19, 23, 19	Conv	32	3	1	19, 23, 19	Conv	64	3	1	19, 23, 19	Conv	64	3	1	19, 23, 19
ELU					ELU					ELU					ELU				
Conv	64	3	1	17, 21, 17	Conv	64	3	1	17, 21, 17	Conv	64	3	1	17, 21, 17	Max Pool		3	2	17, 21, 17
ELU					ELU					ELU					PIF 5x5x5	6	3	1	8, 10, 8
Conv	64	3	1	15, 19, 15	PIF 5x5x5	6	3	1	15, 19, 15	Max Pool		4	3	15, 19, 15	ELU				
ELU					ELU					Linear		100		6144	Linear		100		1458
Max Pool		4	2	13, 17, 13	Linear		100		7776	ELU					ELU				
Linear		100		11200	ELU					Linear		1		100	Linear		1		100
ELU					Linear		1		100										
Linear		1		100															

ADNI

Model A	#Filters	Size	Stride	Input Size	Model A PIF	#Filters	Size	Stride	Input Size	Model B	#Filters	Size	Stride	Input Size	Model B PIF	#Filters	Size	Stride	Input Size
Conv	8	3	1	94, 114, 96	Conv	8	3	1	96, 114, 96	Conv	64	3	1	96, 114, 96	Conv	64	3	1	96, 114, 96
ELU					ELU					ELU					ELU				
Max Pool		3	3	94, 112, 94	Max Pool		3	3	94, 112, 94	Max Pool		3	3	94, 112, 94	Max Pool		3	3	94, 112, 94
Conv	16	3	1	31, 37, 31	Conv	16	3	1	31, 37, 31	Conv	64	3	1	31, 37, 31	Conv	64	3	1	31, 37, 31
ELU					ELU					ELU					ELU				
Max Pool		3	2	29, 35, 29	Max Pool		3	2	29, 35, 29	Max Pool		3	2	29, 35, 29	Max Pool		3	2	29, 35, 29
Conv	32	3	1	14, 17, 14	Conv	32	3	1	14, 17, 14	Conv	64	3	1	14, 17, 14	Conv	64	3	1	14, 17, 14
ELU					ELU					ELU					ELU				
Conv	64	3	1	12, 15, 12	Conv	64	3	1	12, 15, 12	Conv	64	3	1	12, 15, 12	Conv	64	3	1	12, 15, 12
ELU					ELU					ELU					ELU				
Conv	36	3	1	10, 13, 10	PIF 5x5x5	3	3	1	10, 13, 10	Conv	36	3	1	10, 13, 10	PIF 5x5x5	3	3	1	10, 13, 10
ELU					ELU					ELU					ELU				
Max Pool		4	2	8, 11, 8	Linear		100		1134	Max Pool		4	2	8, 11, 8	Linear		100		
Linear		80		1296	ELU					Linear		80		1296	ELU				1134
ELU					Linear		1		100	ELU					Linear		1		
Linear		1		80						Linear		1		80					80

Table A.1: overview of model settings for all models trained on each data set. Model A PIF (model B PIF) uses the architecture from model A (model B) and replaces the last convolutional layer with a PIF layer.

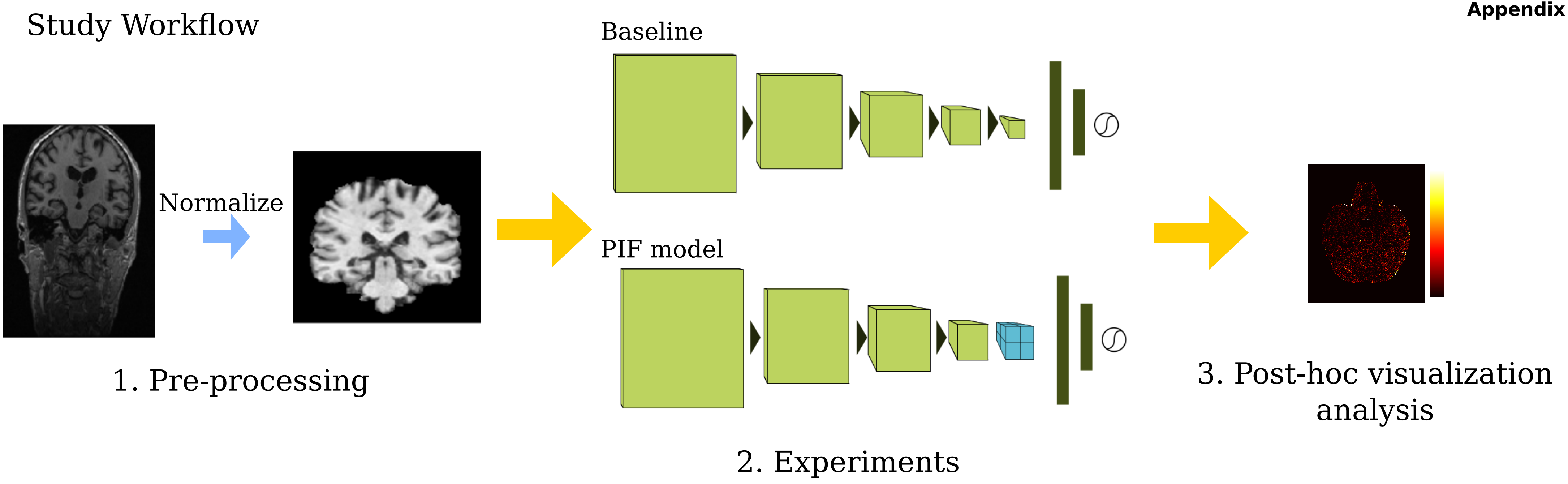

Figure A.1: All MR images are first normalized using non-linear registration and skull stripping. Then, a baseline model is trained and optimized for the data set. The final layer of the baseline model is replaced with a PIF layer and the model is then trained again and compared to the baseline. Finally, post-hoc visualizations have been created using LRP to study the assumptions about the regionality of higher level filters.

Appendix

Receiver operating characteristic curves

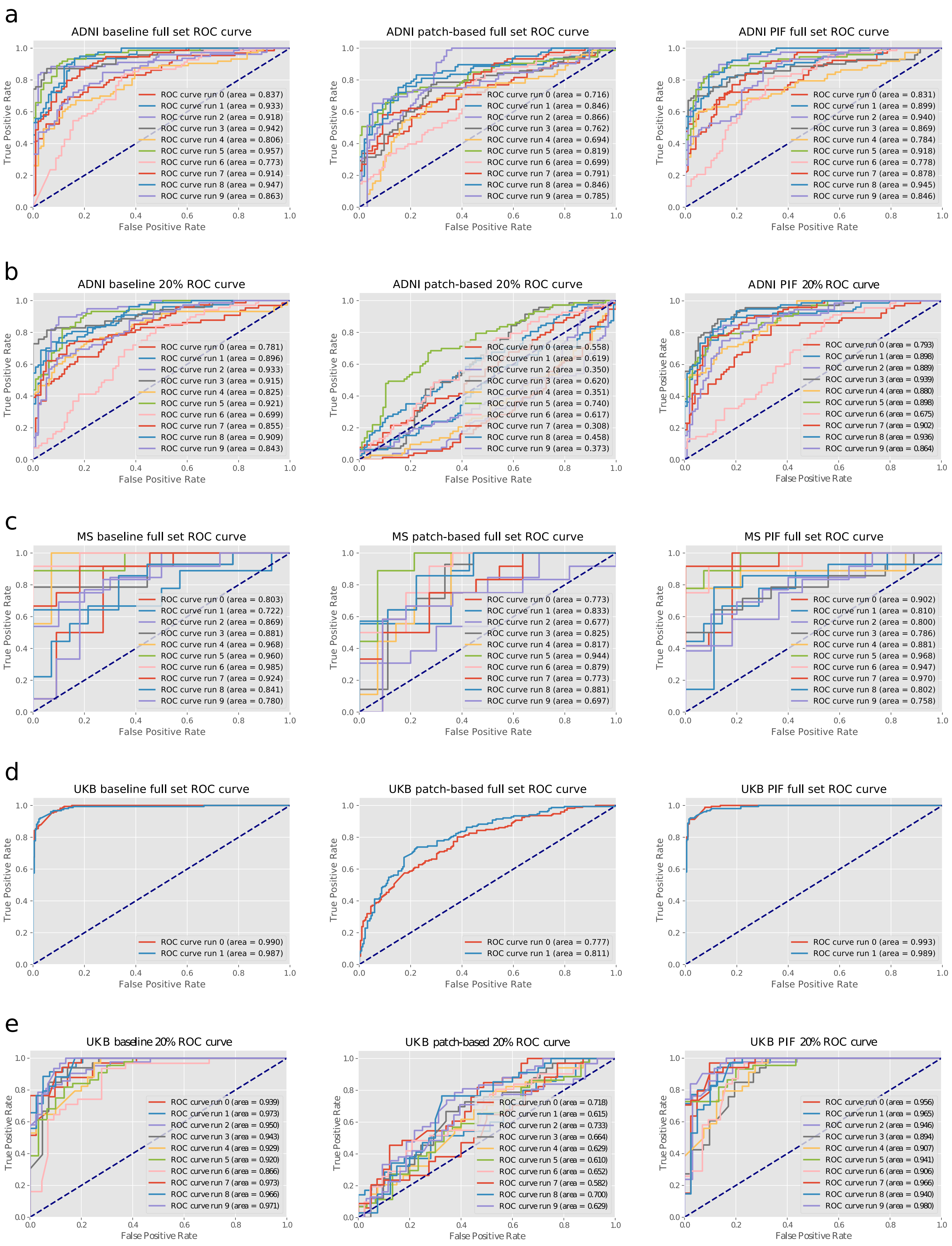

Figure A.2: Receiver operating characteristic curves for all models including all runs and the area under the curve per run.
